# *Drosophila suzukii* Susceptibility to the Oral Administration of *Bacillus thuringiensis*, *Xenorhabdus nematophila* and Its Secondary Metabolites

**DOI:** 10.3390/insects12070635

**Published:** 2021-07-13

**Authors:** Maristella Mastore, Sara Caramella, Silvia Quadroni, Maurizio Francesco Brivio

**Affiliations:** 1 Laboratory of Comparative Immunology and Parasitology, Department of Theoretical and Applied Sciences, University of Insubria, 21100 Varese, Italy; maristella.mastore@uninsubria.it (M.M.); sara.caramella97@gmail.com (S.C.); 2 Laboratory of Ecology, Department of Theoretical and Applied Sciences, University of Insubria, 21100 Varese, Italy; silvia.quadroni@uninsubria.it

**Keywords:** *Drosophila suzukii*, biological control, *Bacillus thuringiensis*, Xenorhabdus nematophila, secondary metabolites, secretion, entomopathogen, bioinsecticides, combined administration, gut damage

## Abstract

**Simple Summary:**

In recent decades, climate change and the international fruit trade have favored the movement of allochthonous species such as harmful insects into new geographic areas. The settlement of phytophagous insects and vectors in new areas, where potential predators are often lacking, has increased the use of chemical insecticides for their control. The intensive use of these substances represents a serious problem for ecosystems and human health; a possible alternative to chemical control is biological control, i.e., the use of biological insecticides that are compatible with the environment. The aim of our work was to further improve biological control methods for the management of the dipteran Spotted Wing Drosophila, an insect recently introduced in America and Europe, which can damage thin-skinned fruit crops. The methodologies applied are based on the combined use of different entomopathogens, i.e., bacteria, fungi, nematodes, etc., harmful for insects, with the purpose of increasing their effectiveness. The results obtained show that the combined use of two entomopathogenic bacteria increases both the lethality and rapidity of action. From an application viewpoint, studies like this are essential to identify new methods and bioinsecticides and, once transferred to the field, can be crucial to eliminate or, at least, reduce the use of chemicals.

**Abstract:**

*Drosophila suzukii*, Spotted Wing Drosophila (SWD), is a serious economic issue for thin-skinned fruit farmers. The invasion of this dipteran is mainly counteracted by chemical control methods; however, it would be desirable to replace them with biological control. All assays were performed with *Bacillus thuringiensis* (Bt), *Xenorhabdus nematophila* (Xn), and Xn secretions, administered orally in single or combination, then larval lethality was assessed at different times. Gut damage caused by Bt and the influence on Xn into the hemocoelic cavity was also evaluated. In addition, the hemolymph cell population was analyzed after treatments. The data obtained show that the combined use of Bt plus Xn secretions on larvae, compared to single administration of bacteria, significantly improved the efficacy and reduced the time of treatments. The results confirm the destructive action of Bt on the gut of SWD larvae, and that Bt-induced alteration promotes the passage of Xn to the hemocoel cavity. Furthermore, hemocytes decrease after bioinsecticides treatments. Our study demonstrates that combining bioinsecticides can improve the efficacy of biocontrol and such combinations should be tested in greenhouse and in field in the near future.

## 1. Introduction

Invasive insect species endanger crops, food supplies, damage urban greenery, compete with native species, and are extremely expensive to manage. Their spread into new areas of the world is caused by the increase of commercial exchanges and tourism, as well as by the changed environmental conditions promoted by climate change.

Among many insect species that have moved from Southeast Asia to the Americas and Europe in the last decade, *Drosophila suzukii* Matsumura (Diptera: Drosophilidae), also named Spotted Wing Drosophila (SWD), is a serious threat to the cultivation and marketing of thin-skinned red fruits in the invaded areas. The high invasiveness of the SWD is based on several factors: the presence of free niches without competitors and natural predators, its ability to adapt to temperate climates, its reproductive efficiency, and its dispersal through the fruit trade. The significant economic damage caused by SWD is also due to the life cycle and feeding behavior of the fly, which attacks ripening healthy fruit and, through oviposition in the flesh of it, causes evident damage just prior to harvest and marketing. Moreover, the damage to the fruit skin also promotes secondary infections by pathogens, such as bacteria, fungi and yeasts, which lead to further deterioration of the fruit [1].

The control of the SWD is performed with various methods based on different strategies. Besides synthetic compounds with insecticidal activity, other methods less invasive for the environment have been tested; among them there are physical barriers, pheromone traps, natural substances with deterrent action, insecticides of natural origin, and biological insecticides. Current efforts for SWD control rely mostly on the use of chemical insecticides. However, in addition to the known regulatory limitations on the use of pesticides, the rapid reproduction rate of the dipteran requires multiple administrations, which can cause a large increase of residues in the fruit, and the emergence of resistance forms [2]. Moreover, the correct use of synthetic pesticides has a moderate effect on the adults, and often their action is limited to the outside of the treated fruit and does not reach the developing larvae which are protected inside the fruit’s flesh.

Possible alternatives to chemical pesticides are natural substances, such as repellents, deterrent to oviposition and toxicants [3], or biological control methods based on bioinsecticides [4]. To date, there are many studies on the use of biological agents to control SWD; pathogenic fungi, bacteria, and nematodes can be sprayed directly on epigeal parts of the plant or to soil to infect adults, larvae, and pupae. These agents are insect-specific and are not harmful to crops and consumers [5].

Currently, only fungal products have been tested in the field, while information on bacteria and nematodes comes mainly from laboratory trials. For all these agents, the reported efficacy varies from no effect to high mortality.

Among bioinsecticides potentially useful against SWD, entomopathogenic nematodes (*Steinernematidae* spp. and *Heterorhabditidae* spp.) and their symbiotic bacteria (*Xenorhabdus* spp. and *Photorhabdus* spp.) can be included. These nematocomplexes at the infective juvenile (IJ) stage can penetrate the target insect inside the infested fruit [6,7]. In some studies, in which immune defenses of the insect target have also been investigated [8,9], nematodes have been shown to be quite effective, although semi-field and field studies are very scarce [10]. Moreover, the isolated bacteria symbionts, *Xenorhabdus* spp. and *Photorhabdus* spp., have been tested for efficacy as bioinsecticides, thanks to the lethality of their secondary metabolites [11,12,13]. These bacteria have been seen to be effective as host killers when they are naturally released by the nematode, but the potential of isolated symbionts has also been investigated in both oral administrations and hemocoelic microinjections, carried out by whole bacteria or their secretions [14,15,16,17].

Several serovars of *Bacillus thuringiensis* (Bt) have been tested on SWD, mostly in laboratory studies, and they have shown variable efficacy, depending on its concentration and have been shown to be mainly lethal on early larval stages of the dipteran [18,19]. Cossentine et al. [18] demonstrated that the Bt serovar kurstaki strain HD-1 is the most effective against the first instar of SWD larvae, and the strain HD-263 showed a high efficacy against second instar larvae at treatment times longer than 7 days.

Since treatments with single bioinsecticides often produce appreciable results only after long periods of time, and it is necessary to obtain a significant mortality in the shortest time of treatment, the use of targeted combinations of more bioinsecticides could represent an improvement of the biocontrol techniques against SWD.

A number of papers described single or combined administrations of nematodes, microorganisms, and secretions, even under different temperature conditions [20,21,22], and, in some cases, the results obtained are positive, i.e., the combinations significantly increase the effectiveness of the biocontrol method. In a previous work [9] we observed that the presence of *Steinernema carpocapsae* combined with Bt subspecies kurstaki, enhanced the lethality of SWD larvae, when administered concurrently or time shifted. The obtained data showed a relevant decrease of the time required to reach a high kill rate, being 16–24 h with concentrations of 0.564 µg/mL and 8 × 10^2^ IJ/mL for Bt and *S. carpocapsae*, respectively.

In this paper, we aimed to investigate the effects of the oral administration of Bt with *Xenorhabdus nematophila* (Xn) isolated from *S. carpocapsae*, or with Xn secretions, on SWD early instars larvae. Firstly, we assayed the lethal effects of individual bioinsecticides administration, then we evaluated a possible increase in efficacy induced by their combination. Moreover, we analyzed the morphology of the host gut to evaluate the possible damage caused by Bt or Xn after the oral uptake; indeed, we hypothesized that this damage could enhance the passage of microorganisms into the host hemolymph. Finally, we analyzed the effect of bioinsecticides on hemolymph cell population.

## 2. Materials and Methods

### 2.1. Chemicals and Instruments

All reagents were supplied by Sigma Chemicals (St. Louis, MO, USA) and Merck Millipore Ltd. (Tullagreen, Cork, Ireland). Instruments were provided by Bio-Rad Laboratories (Detroit, MI, USA) and Euroclone S.p.A. (Milan, Italy, EU). Centrifugations were carried out with a SIGMA 1–14 (SciQuip Ltd., Newtown, Wem, Shropshire, UK) microcentrifuge and Eppendorf 5804 (Eppendorf, AG, Hamburg, Germany) refrigerated centrifuge. Spectrophotometric analysis was performed with a Jasco V-560 spectrophotometer (Easton, MD, USA). All materials, buffers, and solutions were autoclaved or filtered with 0.22 µm Minisart filters (Sartorius, Goettingen, Germany).

For light microscopy and images capture, a SZQ4 stereomicroscope (OPTIKA Italy Srl, Ponteranica (BG), Italy) connected to an OPTIKA C-HP digital camera, was used. For fluorescence microscopy, an Olympus IX-51 microscope, connected to an OPTIKA mod. C-P20CM digital camera and a Leica TCS SP5 confocal laser scanning system (Leica Microsystems GmbH, Wetzlar, Germany) were used.

### 2.2. Target Insect

*D. suzukii* larvae native to Catalonia (NE Spain) were reared on a specific diet [8] and housed in a climatic chamber at 25 °C and 45% relative humidity, with a 12:12 h photoperiod. Only healthy larvae in comparable growth stage (L1) were used for mortality trials and bioassays. The same housing conditions were maintained during all tests.

### 2.3. Bacterial Strains and Cultures

Entomopathogenic bacteria, *B. thuringiensis* subspecies kurstaki (strain EG 2348), *X. nematophila* isolated from *S. carpocapsae* (strain B14) according to the method by Park and Kim [23], and *X. nematophila* secretions, were used for assays. Bt and Xn were inoculated on Luria-Bertani (LB) and Tryptic Soy (TSB) broths, respectively. Bacterial cultures were grown for 24 h under shaking at 30 °C, in dark condition. Bacterial growth was verified by spectrophotometric measurement of biomass (λ = 600 nm).

Bt spores and crystals were isolated as described by Cossentine et al. [18] and their presence was monitored by phase contrast microscopy. Before the use at the concentration of 0.564 µg/mL, spores and crystals were freeze-dried and resuspended in filtered sterile tap water.

Xn culture was centrifuged at 1700× *g* for 15 min at 20 °C, and bacterial pellet and supernatant broth were recovered. Before use, the pellet was washed twice with fresh medium and the bacterial concentration was determined as colony forming unit (CFU) on agar, by serial dilution method. A supernatant containing Xn secretions, obtained from a 10^9^ CFU/mL culture, was centrifuged several times, filtered on 0.22 µm Minisart filters to remove any contaminants, and finally dialyzed against 10 mM Tris HCl, pH 7.4. The protein concentration of Xn secretion was detected by Bio-Rad reagent (Bio-Rad protein assay, Cat. 5000006) according to manufacturer protocol.

*Escherichia coli* (strain D31) used for gut morphology analysis, was inoculated into LB broth and incubated at 37 °C overnight under shaking. Bacterial growth was checked, and cells were processed by centrifugation at 1700× *g*, for 15 min at 20 °C. The bacterial pellet was recovered and washed several times with PBS buffer (pH 7.4). *E. coli* were conjugated with fluorescein isothiocyanate (FITC), following a modification of the method developed by Hazenbos et al. [24]. Bacteria were immediately used or stored at 20 °C.

### 2.4. Oral Administrations: Single and Combined Treatments

To verify the effects of various bioinsecticides on SWD larvae, the agar trap method was used as described by Mastore et al. [9]. Larvae were passively fed on substrate, and susceptibility was evaluated by a single oral administration of spores and crystals of Bt and Xn bacteria, and Xn secretions. A preliminary assay was performed to assess the mortality of the larvae treated with different concentrations of Xn (1 × 10^5^, 1 × 10^7^, 1 × 10^9^ CFU/mL), and mortality was monitored under a stereomicroscope at 24 and 48 h after treatment. When the efficacy of Xn was evaluated, single oral administration assays of the various bioinsecticides, followed by combined treatments with Bt/Xn or Bt/Xn secretions, were performed. Larvae mortality was monitored at 16, 24, 32, and 48 h after each treatment. In all assays, only the larvae that lacked movement and did not pupate were considered dead.

One ml of each bioinsecticide was added to a Petri dish containing 20 larvae. For single administrations, Bt was used at the concentration of 0.564 µg/mL (corresponding to a density of 5.64 × 10^–2^ µg/cm^2^), Xn was added at 1 × 10^9^ CFU/mL and Xn secretions were used at the concentration of 10 mg/mL. For combined administrations, the same concentrations of Bt/Xn or Bt/Xn secretions were used. The bioinsecticides were administered concurrently (t_0_), or with the second treatment, with Xn or Xn secretions, delayed by 16 h after the treatment with Bt. Control assays were carried out by incubating SWD larvae with 1 mL of medium (LB or TSB) without bioinsecticides.

### 2.5. SDS-PAGE Analysis of X. nematophila Secretions

We analyzed the protein patterns of Xn secretions using the electrophoretic separation by SDS-PAGE [25]. Secretions were obtained as described in paragraph 2.3 and, before electrophoretic separation, were dialyzed against Tris-HCl overnight at 4 °C. Samples were fractioned by Amicon^®^ Ultrafilters (Millipore, Burlington, MA, USA) cut-off 30 KDa and precipitated with trichloroacetic acid (20% *V*/*V*). Then, the samples were resuspended in 1× SDS sample buffer, denatured for 5 min at 100 °C, and 1 µg/well was loaded. TSB broth was processed in the same way and used as control. Electrophoresis was carried by a vertical PROTEAN^®^ II xi Cell (Bio-Rad) at 50 V (constant voltage) overnight. Protein patterns were detected by Silver Staining.

### 2.6. Hemolymph Collection and Total Hemocytes Count (THC)

Total hemocytes count (THC) was carried out after larvae treatments with single administration of Bt, Xn or Xn secretions. SWD early instar larvae were incubated for 24 h with bioinsecticides and then hemolymph was extracted. Before extraction, the larvae were washed in 70% ethanol, sterile PBS, and then anesthetized at 4 °C. Hemolymph from five larvae was collected by injuring the pharyngeal-ventral region with microsurgical forceps. The larvae were transferred into PCR tubes properly prepared for the procedure as described by Garriga et al. [8]. The tube was inserted into a 0.5 mL Eppendorf containing 10 µL of Schneider medium, then the samples were centrifuged at 250× *g* for 5 min at 4 °C, and hemolymph with hemocytes was collected in the larger tube. Hemocyte suspension was quantified using a Corning Cell counter (Corning incorporated, life sciences, Tewksbury, MA, USA) at magnification 5×, and the data obtained were processed by CytoSmart^®^ software. Three repetitions were carried out for each treatment; control was performed with not-treated larvae.

Cell-free hemolymph (CFH) was obtained from 800 larvae; hemolymph was processed by centrifugation at 250× *g* for 5 min at 4 °C in the presence of few phenylthiourea crystals to avoid unwanted activation of phenoloxidase. Supernatant (CFH) was collected and filtered through 0.22 µm Minisart filters, then inactivated at 60 °C for 2 h in a thermostatic bath to prevent any enzymatic activation. CFH samples were processed by high-speed centrifugation to remove any contaminants, and then added to the bacterial culture media.

### 2.7. Interference of Bioinsecticide Secretions on Bacterial Growth

We tested whether Bt or Xn secretions could affect mutual growth in combined administration. Xn and Bt secretions were obtained from culture media as described in Section 2.3 or, to simulate the growth conditions in the hemocoelic cavity, with the addition of 4% CFH to the media. Before use, all secretions were filtered and dialyzed to remove any contaminants.

Aliquots (50 µL) of Bt or Xn secretions, obtained with or without hemolymph, containing 10 µg/mL of proteins, were added to 50 µL of a 10^4^ and 10^5^ CFU/mL bacterial suspension of Xn and Bt, respectively, obtained by serial dilutions with appropriate culture medium (the dilution used depends on the growth rate and stage of the strain).

Samples, i.e., Xn plus Bt secretions, Bt plus Xn secretions, both obtained with or without CFH, and Bt alone or Xn alone, were incubated for 3 h at 30 °C under shaking. After incubation, 100 µL of each sample was plated on solid agar (LB or TSB). To determine the viable cell count, CFU were counted after incubation at 30 °C overnight. Bacterial growth was defined as the percentage of bacterial survival relative to the control (bacterial suspension incubated without secretions). To determine CFU/mL, the average colony count was determined by the following formula: CFU/mL = average colony count/dilution factor.

### 2.8. Analysis of the SWD Larvae Gut Morphology

The gut morphology of SWD larvae (L1 stage) was observed after treatment with dyes (Erythrosine, TRITC-Dextran 70kD), *E. coli*-FITC, and the bioinsecticides Bt or Xn-GFP.

Treatments were performed using agar trap with (0.564 µg/mL) or without Bt and incubating larvae at 25 °C in the climate chamber for 3 and 12 h. As controls, 4 mg/mL erythrosine or 200 µL TRITC-Dextran (1 mg/mL) were added to the agar trap. The various treatments with bacteria were performed by supplementing the substrate with 200 µL of *E. coli*-FITC (10^9^ CFU/mL), Bt plus 200 µL of TRITC Dextran (1 mg/mL), 200 µL of Xn-GFP (10^9^ CFU/mL), and Bt plus 200 µL of Xn-GFP (10^9^ CFU/mL).

After treatments, larvae were washed with physiological buffer, then placed on a slide and covered with the coverslip; observations were made with fluorescence, light, or confocal microscopes.

### 2.9. Data Analysis

All data on single and combined administrations were reported as mean plus standard deviation of six replicates. We applied the two-way analysis of variance (ANOVA) considering treatment and time as variables and the percentages of SWD mortality as observations. Before the ANOVA, the percentage data were arcsin-transformed. After the ANOVA, we used the Tukey HSD post-hoc test to detect possible significant (*p* < 0.05) differences of insect mortality between different treatments at the same time and between times within the same treatment.

Data on bacterial growth and THC were reported as mean plus standard deviation of three replicates. We applied the one-way ANOVA followed by the Tukey HSD post-hoc test to detect possible significant (*p* < 0.05) differences of the growth of Bt and Xn due to the possible interference of their respective secretions, and of THC due to the effects of single bioinsecticides (Bt, Xn, and Xn secretions). The statistical analyses were performed using free software PAST 3.09 (https://www.nhm.uio.no/english/research/infrastructure/past/, accessed on 15 December 2019).

## 3. Results

### 3.1. Bioinsecticides Administration

**Figure 1:**
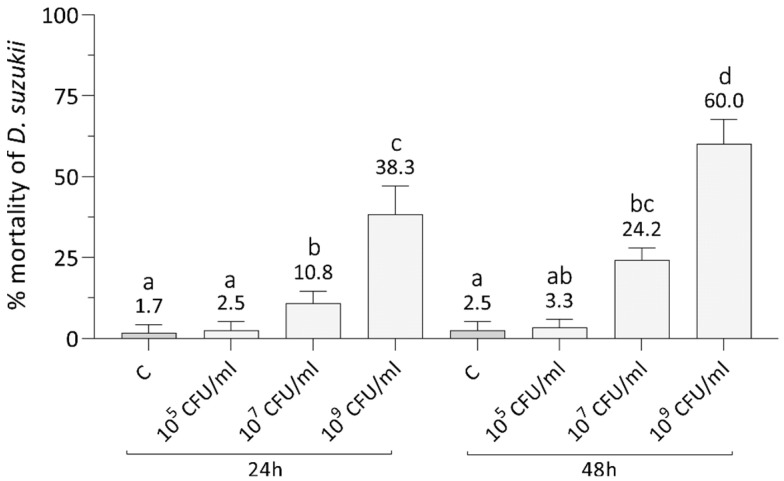
Effects of the oral administration of various concentrations of *X. nematophila* to L1 larval stages of SWD. A marked increase of mortality compared to the control (C) was observed only with a bacterial load of 10^9^ CFU/mL, causing a mortality higher than 50% at 48 h. Letters (a–d) indicate significant differences in the pairwise comparisons (Tukey test, *p* < 0.05, see Table S1).

As a preliminary assay, we assessed the effects of increasing concentrations of Xn orally administered by agar trap to early instars SWD larvae (Figure 1). Two-way ANOVA highlighted significant differences of SWD mortality due to both concentration (F_3,48_ = 123, *p* < 0.001) and time (F_1,48_ = 18, *p* < 0.001), but the interaction between these two factors was not significant (F_3,48_ = 3, *p* = 0.051). Treatments with Xn concentration of 10^9^ CFU/mL showed a larval mortality rate significantly different from the control and the other concentrations (Tukey test, *p* = 0.0001, Table S1), as high as 60% on average after 48 h. There was also a significant mortality rate at 24 h (Tukey test, *p* = 0.0001, Table S1), but it did not exceed 38% on average. The administration of concentrations below 10^9^ CFU/mL (10^7^ and 10^5^ CFU/mL) led to negligible mortality rates at both time intervals considered. The lowest concentration caused a mortality not significantly different from the control (Tukey test, *p* > 0.05, Table S1). The middle concentration (10^7^ CFU/mL) determined a significantly higher mortality than the control (Tukey test, *p* < 0.01, Table S1) but of only 24% on average at 48 h.

**Figure 2:**
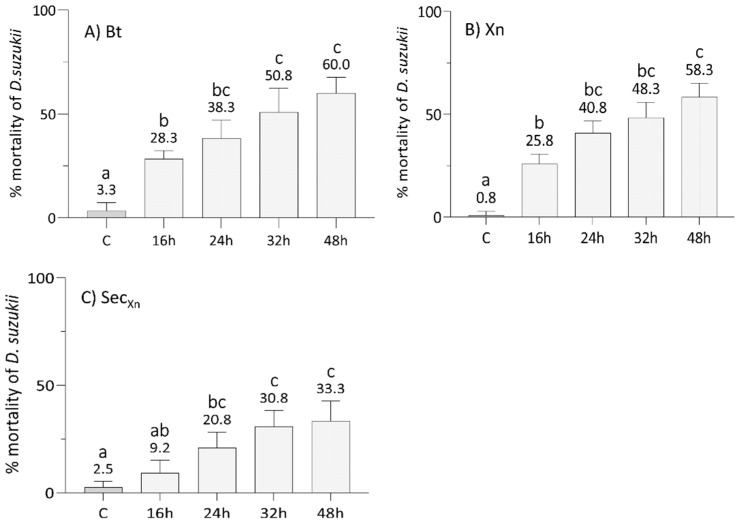
Effects of the administration of *B. thuringiensis* (**A**), *X. nematophila* (**B**), and *X. nematophila* secretions (**C**) at 16, 24, 32, and 48 h, to L1 larval stages of SWD. The efficacy of Bt (0.564 μg/mL) and Xn (10^9^ CFU/mL) was quite comparable, leading to a mortality of approximately 60% at 48 h. Administration of *X. nematophila* secretions (10 µg/mL) resulted instead in a lower mortality (approximatively halved at 48 h) compared to the bacteria. C: Control at 48 h. Letters (a–c) indicate significant differences in the pairwise comparisons between different times within the same treatment (Tukey test, *p* < 0.05); see Table S2 for detail and other comparisons.

**Figure 3:**
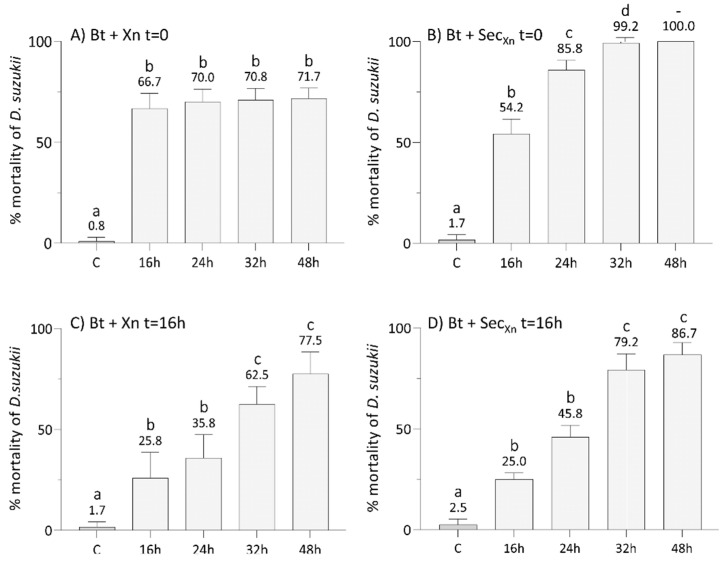
Lethal effects on SWD larvae of combined administration of *B. thuringiensis* plus *X. nematophila* (panels **A** and **C**), or plus *X. nematophila* secretions (panels **B** and **D**), either simultaneously at t = 0 (panels a and b) or with the second treatment delayed by 16 h (panels c and d). All the assays with combined administration resulted in a higher and faster mortality compared to the single treatments (see Figure 2). Concurrent administrations were more effective than time-shifted administration. C: control at 48 h. Letters (a–d) indicate significant differences in the pairwise comparisons between different times within the same treatment (Tukey test, *p* < 0.05); see Table S2 for detail and other comparisons.

After an assessment of the optimal concentration of Xn, we tested the efficacy of single bioinsecticides, i.e., Bt, Xn, and Xn secretions, at 16, 24, 32, and 48 h (Figure 2), and then of the combinations of Bt plus Xn and Bt plus secretions of Xn to evaluate whether their concurrent administration (Figure 3A,B) or with a delay of 16 h of the second bioinsecticide, i.e., Xn and its secretions (Figure 3C,D), could improve the mortality rate and reduce the treatment time. A significant effect of treatment (F_7,192_ = 204, *p* < 0.001), time (F_3,192_ = 111, *p* < 0.001) and the interaction between treatment and time (F_21,192_ = 7, *p* < 0.001), was detected by two-way ANOVA.

The comparison of the data from Figure 2A (Bt) and Figure 2B (Xn) highlighted similar effects (Tukey test, *p* > 0.05, Table S2) of the two entomopathogenic bacteria at concentrations of 0.564 µg/mL for Bt and 10^9^ CFU/mL for Xn, with a time-dependent lethality rate on average (Table S2). However, with both microorganisms, SWD mortality did not exceed 60% at 48 h. The results obtained with concentrations of 10 µg/mL of Xn secretions (Figure 2C) led to a mortality, increasing over time on average (Table S2), with a peak of 33% 48 h post treatment. In this case, the mortality rates were significantly lower compared to those observed for both the bacteria (Bt and Xn) (Tukey test, *p* < 0.05, Table S2).

The graph in Figure 3A shows that concurrent treatment of Bt with Xn, resulted in a significant increase in the mortality rate compared to single administrations (Tukey test, *p* < 0.05, Table S2) that, after 16 h, remained almost constant (67–72%) until 48 h.

The results obtained by the concurrent combination of Bt with Xn secretions (Figure 3B) showed a significantly higher efficacy from 24 h after treatment (Tukey test, *p* < 0.05, Table S2), leading to a mortality rate of 100% after 48 h. When treatments with Xn (Figure 3C) and its secretions (Figure 3D) were delayed by 16 h after Bt administration, in both cases the mortality rate increased over time reaching 78% and 87% within 48 h, respectively, but never reaching 100%. The time-shifted treatments (Xn or secretions administered 16 h after Bt) were significantly less effective than the concurrent treatments (bioinsecticides administered at *t* = 0), but more effective than single administrations (Tukey test, *p* < 0.05, Table S2).

### 3.2. Secretion Protein Pool of X. nematophila

The proteic content of Xn secretions obtained from 24 h culture broth was analyzed by SDS-PAGE.

**Figure 4:**
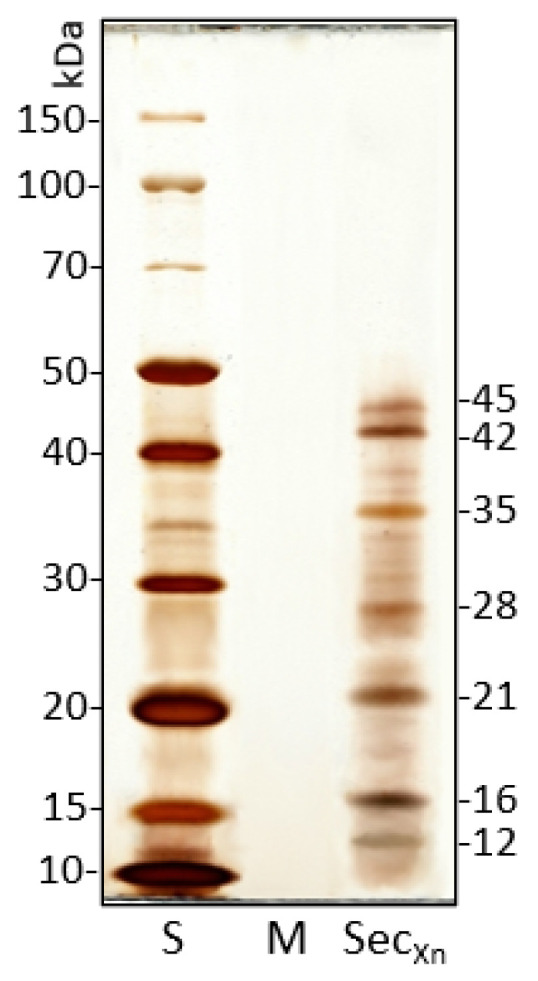
SDS-PAGE pattern of *X. nematophila* secretions shows 7 main bands, ranging from 10 to 45 kDa, and some less represented bands. The bacteria medium (M) has no detectable protein content. S: standard molecular weights, M: culture medium, Sec_Xn_: *X. nematophila* secretions (1 μg/well loaded).

The electrophoretic pattern shows the presence of seven main bands in the range 10–45 kDa and some less represented bands (Figure 4). In the range of molecular weights observed by PAGE, various bands are present and could include putative *Xenorhabdus* secreted factors with biological activity such as serine proteases, metallo-proteases, and protease inhibitors, as reported by NCBI/Protein (https://www.ncbi.nlm.nih.gov/protein/, accessed on 10 June 2021).

### 3.3. Mutual Interference of Secretions on Bacterial Growth

**Figure 5:**
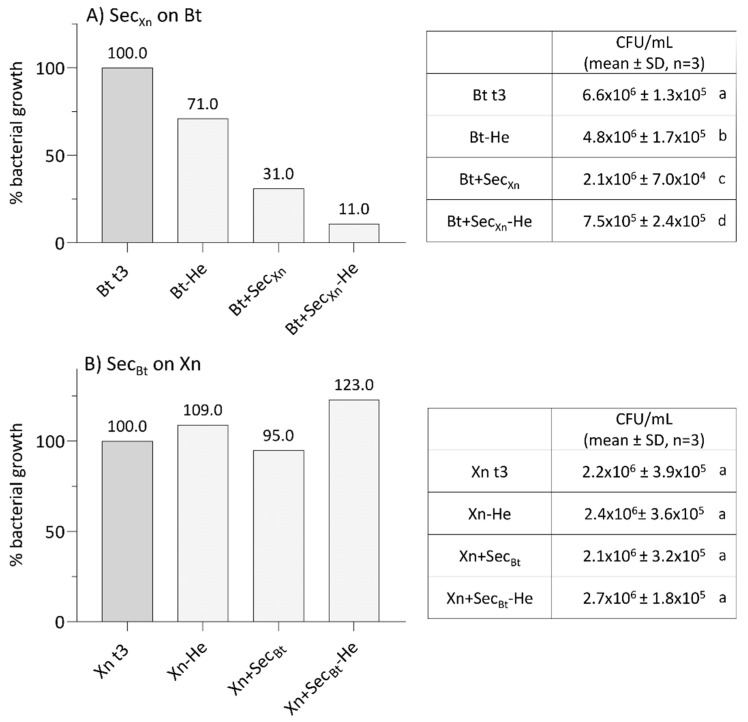
Analysis of bacterial growth of *B. thuringiensis* in the presence of *X. nematophila* secretions (**A**), and of *X. nematophila* in the presence of *B. thuringiensis* secretions (**B**), either in the presence or absence of SWD hemolymph. Bacteria were incubated for 3 h in the absence (panel A, Bt t3 and panel B, Xn t3) or in the presence of secretions (panel A, Bt+Sec_Xn_ and panel B: Xn+Sec_Bt_). Bacteria were also treated with secretions obtained from cultures grown in the presence of hemolymph CFH (panel A: Bt-He and Bt+Sec_Xn_-He; panel B: Xn-He and Xn+Sec_Bt_-He). In the assays the interference on the growth was observed only on Bt when in the presence of Xn secretions (panel A). Letters (a–d) indicate significant differences in the pairwise comparisons (Tukey test, *p* < 0.05).

After combined treatments of Bt with Xn, we evaluated whether their secondary metabolites, normally secreted during infection, could interfere with the growth and thus reduce their effectiveness. Accounting for the data (Figure 5A), it is evident that Xn secretions, obtained in the presence or absence of CFH from SWD larvae, drastically interfered with Bt growth (One-way ANOVA, F_3,12_ = 755, *p* < 0.001). When secretions were added at a concentration of 10 µg/mL, Bt growth significantly decreased to 31% (Bt + Sec_Xn_), or to 11% in the presence of the host hemolymph (Bt + Sec_Xn_-He), compared to the controls (Bt t3 and Bt-He) (Tukey test, Q = 20–61, *p* = 0.0002). Conversely, Bt secretions did not interfere with Xn growth (Figure 5B; Xn + Sec_Bt_ and Xn + Sec_Bt_-He) (One-way ANOVA, F_3,12_ = 2, *p* = 0.194); the presence of the host CFH even led to an increase of Xn growth, even if not significant.

### 3.4. Effects of Entomopathogenic Bacteria on SWD Gut

**Figure 6:**
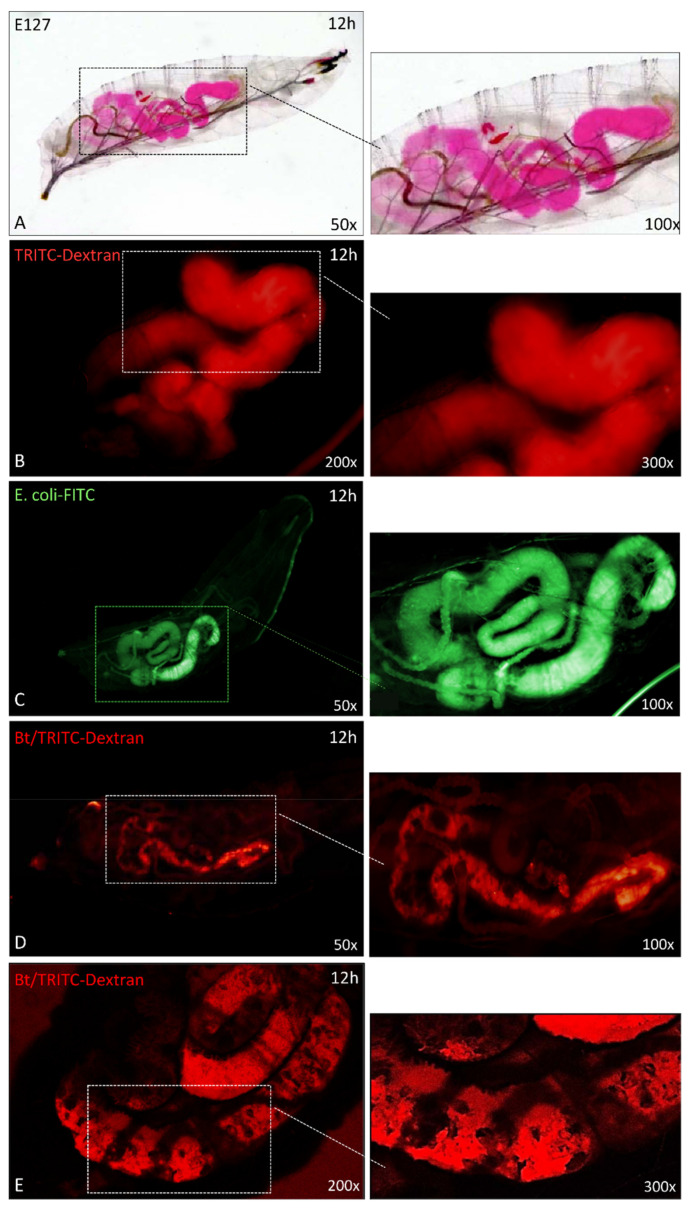
The morphology of SWD gut (L1 larval stages) after oral administration of Erythrosine (E127) (**A**), Dextran-TRITC (**B**), *E. coli*-FITC (**C**), and *B. thuringiensis* plus TRITC-Dextran (**D**,**E**). Panels A and B show the anatomy of healthy larvae fed on Agar-Erythrosine or TRITC-Dextran; the staining highlights the morphology of the whole gut in healthy larvae. A regular morphology was also observed after ingestion of non-entomopathogenic bacteria *E. coli* FITC-conjugated (panel C). Panels D, E: the altered morphology of SWD gut after administration of *B. thuringiensis* plus TRITC-Dextran. After *B. thuringiensis* treatments the damage of the gut wall was highlighted by the presence of TRITC-Dextran. Panel E is a confocal image of larvae after Bt/dextran treatment. Insets at right are enlargements of the micrographs.

In order to explain the increase in efficacy observed by combined administrations, we analyzed the morphology of the gut of SWD larvae, 3 and 12 h after treatments (Figure 6).

The anatomy of the apparatus of healthy larvae was observed using two tracers, i.e., erythrosine (E127) and TRITC-Dextran; the correct morphology of the gut can be observed in Figure 6A,B. Moreover, we analyzed the gut morphology in the presence of a non-pathogenic bacterium, *E. coli* FITC-conjugated (Figure 6C), the micrograph shows the correct anatomy of the organ without damages. Figure 6D,E show the damage caused by Bt treatment at 12 h: severe lesions of the intestinal wall can be observed thanks to the fluorescence of TRITC-dextran (Figure 6D); the organ shows a shrinkage with large, damaged areas, particularly evident in the micrograph of Figure 6E acquired by confocal microscopy.

**Figure 7:**
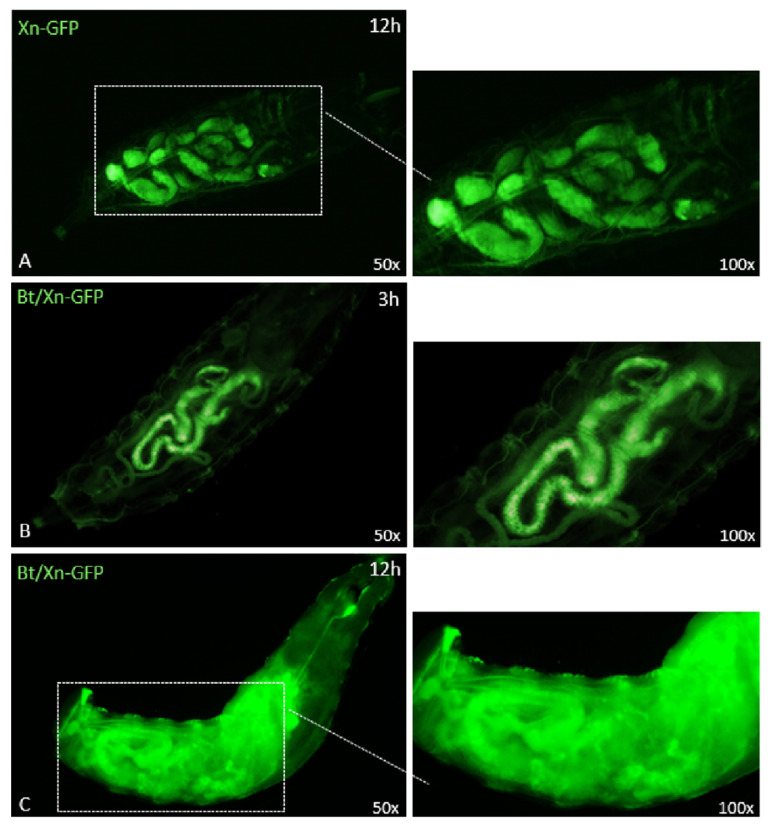
Oral ingestion of *X. nematophila*-GFP in the presence or absence of pre-treatment with *B. thuringiensis* (panels **A**, **B** and **C**). Panel **A** shows the presence of *X. nematophila* in the larvae gut after 12 h of treatment. Panel **B** shows the presence of *X. nematophila* in the gut administered with *B. thuringiensis*, 3 h after oral intake. Panel **C** shows that, after 12 h of treatment with *B. thuringiensis*, *X. nematophila*-GFP was no longer confined within the gut but reached and spread into the hemocoel cavity of the larvae. Insets at right are enlargements of the micrographs.

Moreover, we verified whether the damage caused by Bt toxins could promote the spread of entomopathogenic bacteria into the hemocoel cavity of the insect (Figure 7).

The distribution of GFP-expressing Xn in the intestinal area of larvae not treated with Bt could be observed 12 h after treatment (Figure 7A); no spreading of the bacterium was detected in the hemocoelic cavity. Conversely, when Xn-GFP was taken up by larvae together with Bt, no diffusion was observed 3 h after treatment (Figure 7B); although at 12 h, eased by the injuries, the presence and spread of Xn in the whole hemocoel cavity were clearly detected.

### 3.5. Effects of Bioinsecticides on Total Hemocytes Count (THC)

After the combined administration of Bt and Xn to SWD larvae, Xn diffusion into the hemocoelic cavity was observed due to the damage to the intestinal wall epithelium.

**Figure 8:**
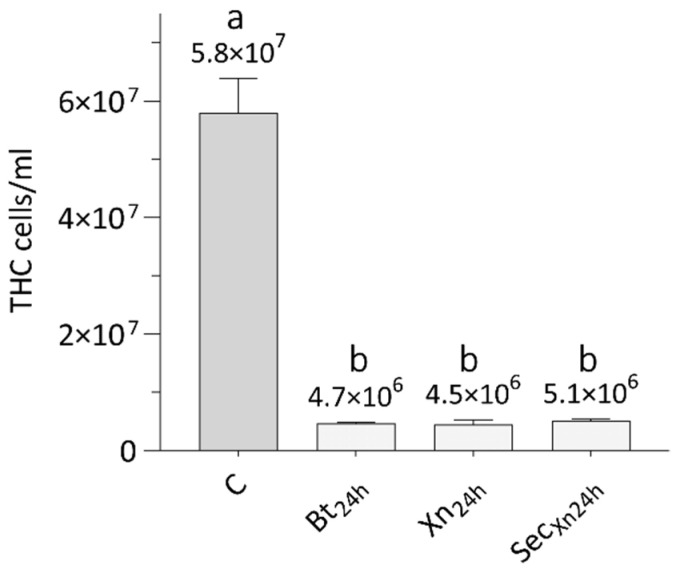
Total hemocytes extracted from naive larvae (C), from larvae treated with *B. thuringiensis* (Bt_24h_), *X. nematophila* (Xn_24h_) and *X. nematophila* secretions (Sec_Xn24h_), 24 h after administration. THC: total hemocytes count. Letters (a–b) indicate significant differences in the pairwise comparisons (Tukey test, *p* < 0.05).

Therefore, we investigated whether the treatments with the single bioinsecticides (Bt, Xn, or Xn secretions), could affect the hemolymph cell population of SWD. The graph in Figure 8 shows that, 24 h after treatment with Bt, Xn, and Xn secretions, the number of cells collected from the larvae underwent a significant similar reduction (Bt_24h_, Xn_24h_, Sec_Xn24h_), compared to the number of hemocytes extracted from naive larvae (C) (One-way ANOVA, F_3,12_ = 236, *p* < 0.001; Tukey test, Q = 31, *p* = 0.0002).

## 4. Discussion

In a previous paper, we evaluated the effects of the administration of the nematode *S. carpocapsae* with Bt either singularly or combined to SWD larvae [9]; the results of the combined administration, when compared to the treatments with single bioinsecticides, revealed a significant increase in effectiveness both in terms of larval mortality rate and reduction in treatment time. The best results were obtained when the larvae were treated with the two entomopathogens simultaneously. Thus, considering the promising previous assays, we decided to further extend the combination protocols by using two microbial pathogens, *B. thuringiensis* and *X. nematophila*.

Bt, when orally assumed, exerts its action in the host gut: bacterial spores produce some toxic compounds, such as the δ-endotoxins, crystal (*Cry1*) and cytolytic (*Cyt*) toxins, which are activated by proteases and the alkaline pH of the insect’s gut [26]. These toxins specifically bind to membrane receptors of the gut epithelium, and this interaction leads to the formation of pores that alter cell permeability, induce cell apoptosis and, consequently, damage the intestinal wall [27]. The injured epithelium allows the passage of Bt spores and could enable the spread of other bacteria of the insect microbiota, to the host hemocoel cavity. When in the hemolymph, Bt transits to the vegetative phase, proliferates and kills the host by septicemia [28,29].

As is already known, Xn is normally released in the host hemolymph by *S. carpocapsae*, which acts as a transporter, and, in the early phase of infection, plays a role in the processes of depression of target insect immune response [30,31,32,33]. As suggested by various authors, Xn also interferes with the immune system by means of its secondary metabolites and surface compounds [16,17,34,35,36]. Xn inhibits the synthesis of eicosanoids affecting cell-mediated processes [15,37], and, besides this, like Bt, Xn can damage the host intestine [38,39,40]. In the late phase, the symbiotic bacterium proliferates in the hemolymph and is responsible for the lethality induced by these nematocomplexes thanks to its toxic secondary metabolites [11,41].

Even if usually Xn is delivered by the nematode, the administration of the isolated symbionts or their secretions, may represent a good starting point to develop new biological control protocols. In addition, the isolation and lab culture of symbionts are relatively simple: by microbiological techniques it is possible to obtain large quantities of bacteria and their secondary metabolites that could be tested by themselves as bioinsecticides [42,43].

Some reviews summarize the potential of isolated Xn or Bt in the biological control of vectors and insect species harmful to agriculture [18,44,45,46,47]. However, the efficacy of these pathogens, when used in single, does not always provide satisfactory results; in addition, their efficacy often requires high concentrations of the pathogen, which can interfere with the life cycle and affect other beneficial organisms present in the environment [48,49]. The reduction of the administered concentrations and the improvement of the rapidity of action, could be achieved by combining different bioinsecticides, thus by exploiting the possible synergistic or additive potential of the applied pathogens [9,50,51,52].

In order to further investigate this hypothesis, in this work we first evaluated the lethal properties of the pathogens Xn and Bt, administered individually. Our results show that both bacteria have a moderate effect on early-stage larvae of SWD; using concentrations of 10^9^ CFU/mL for Xn and 0.564 µg/mL for Bt, mortality rates not exceeding 60% were obtained 48 h post-treatment. It can be deduced from current literature that the results of such assays are variable [9,14,18,53], depending on several factors, such as the target species and their instar, the time of treatment, the concentrations used, and the mode of administration. However, our data agree with those of Park et al. [21] who reported the oral toxicity of Xn on the dipteran *A. aegypti,* leading to 52% of lethality 72 h after ingestion, with a bacterial load of 10^8^ CFU/mL. Effects of Xn and Bt were also observed in larval stages of *Plutella xylostella;* both entomopathogenic bacteria caused significant mortality to the first instars of this lepidopteran, when supplied with diet for 24 h; virulence decreased significantly on fourth larval instars, in particular, Bt induced no significant mortality and treatment with Xn showed no effects [50].

Although a limited number of papers deal with efficacy of Xn, many studies have been carried out on its secondary metabolites; for a review see Dreyer et al. [43]. We therefore isolated and analyzed secretions obtained from the pathogen culture broth. The electrophoretic pattern shows the presence of a protein pool with a molecular weight range between 10 and 45 kDa, in which compounds specifically affecting the intestinal epithelium were identified [40,54]. However, secretions obtained from Xn, administered at a concentration of 10 µg/mL, caused a SWD mortality rate that did not exceed 33% after 48 h. This relatively low efficacy could be due to the oral administration, and thus to the amount of food containing the secretions, which the larvae ingest.

By concurrently administering the combination of Bt and Xn, we observed that the mortality of SWD larvae increased by approximately 10%; the most interesting effect was the reduction in the time required to obtain a significant lethality, a mortality rate of 67% was already recorded at 16 h. The combination of Bt with Xn secretions resulted in a marked increase in mortality, which reached 86% at 24 h, and 100% at 48 h. Overall, the observed increases in efficacy using combinations of bioinsecticides agree with the literature [52,55,56]. In particular, our results are similar to those obtained with the combination of Bt and Xn when used on the dipteran *Aedes albopictus, Culex pipiens pallens* and on the lepidopteran *P. xylostella* [21,50,57].

The different effects observed in our assays with the two combinations, can be explained by the proliferation of Xn cells that, producing secondary metabolites during the infection, inhibit the growth of other microorganisms and interfere with the growth of Bt. Therefore, after the additive action due to both pathogens, the effect of Bt could be attenuated by the presence of the symbiont. However, differences in the natural habitat of the two bacteria need to be accounted for: Bt acts mainly in the gut of the host, while Xn is normally carried and released in the hemolymph; in fact, bacterial growth tests show that Xn grows optimally in the presence of hemolymph, while the presence of hemolymph has an inhibitory effect on Bt. The marked growth of Xn in the presence of hemolymph, can also be explained by its known inhibitory properties on insect immune responses.

The results obtained using Bt with Xn secretions are time-dependent, and Xn metabolites do not appear to interfere markedly with Bt growth, because their concentration is reduced by the intake route, i.e., by feeding. The mortality rate of SWD larvae after 48 h is complete, probably also due to the inhibitory processes of Xn metabolites on the eicosanoid pathway of host hemocytes; the interference with this pathway reduces the potential of humoral and cell-mediated responses [58,59]. Furthermore, these processes occur in the hemocoelic cavity of the insect, and intestinal injuries induced by Bt and Xn secretions, promote and accelerate the passage of bioinsecticides and host microbiota, into the hemolymph.

The hypothesis of a harmful action of Bt on the gut of SWD was confirmed by morphological analyses; the structural integrity of the gut was observed by using different tracers and non-pathogenic fluorescent bacteria (*E. coli*), while the organ alteration was assessed after Bt administration and detection with TRITC-Dextran. The evident gut lesions may represent a rapid route for other microorganisms to enter the hemocoelic cavity, and this was confirmed by observations after treatment with Bt plus Xn-GFP, in which the spread of Xn into the host hemocoelic cavity was evident.

The presence of Bt, Xn or its secretions, in the SWD hemocoel, also drastically affects the immunocompetent cells; in fact, the data obtained show a significant reduction in the population of hemocytes, which leads to a general immunodepression, increasing the efficacy of the bioinsecticides.

Because Xn secretions may interfere with the action of Bt, we planned the assays with a temporal shift in administration; specifically, we administered Bt at t = 0, and 16 h later we added Xn or its secretions. The delay in the addition of Xn could allow Bt to perform its action on the host intestine and, when in the hemolymph, proliferate without the interference from the other pathogen. When Xn was added 16 h after Bt, a marked mortality was observed 32 h post treatment, reaching 62%, and finally 78% at 48 h; the delayed treatment with Xn secretions resulted instead in a mortality rate of 79% at 32 h and 87% at 48 h.

Summarizing the data obtained from the oral administrations, we can suggest that the most effective protocol is the concurrent administration of bioinsecticides, given the rapidity of action, which, in a short time frame, leads to a significant mortality of the SWD larvae.

## 5. Conclusions

The increasing demand for safer control methods for humans and the environment is fueling research on the application of bioinsecticides for pest control. Unfortunately, the use of conventional biological control often provides less definitive results compared to the use of chemicals. Thus, it is necessary to search for innovative methods based on bioinsecticides, possibly by exploiting their individual features that can act synergistically or additively, to improve the effectiveness of these techniques.

To date, research on bioinsecticides has been focused on two main threads: in field or green-house studies, and laboratory studies; in the first case the results may appear more relevant and immediately useful, but the application of these techniques in the field always needs in-depth a priori studies that analyze the interactions between parasites or pathogens and their target organisms at the biological level. The knowledge of the processes that regulate the physiological interactions between entomopathogens and insects is the basic starting point for the understanding and improvement of biological control methods.

Our study can be included in this framework, and our results obtained in the laboratory show that biological control techniques carried out through combinations of bioinsecticides can markedly improve their action, and so their effectiveness, and could be later applied in field trials. A possible use of these techniques is conventional spraying, by which bioinsecticides reach the flesh of the fruit in the areas injured by the female ovipositor. Contact of the pathogens with the pests could be facilitated by the limited penetration depth of the early-stage larvae into the fruit flesh. In addition, as with many formulations used for *B. thuringiensis*, a suitable medium should be appropriately developed, the requirements of which should be compatible with the physiology of the two pathogens and should be persistent in the presence of changes in environmental conditions.

## Data Availability

The data presented in this study are available upon request to the corresponding author.

## Supplementary Materials

The following are available online at https://www.mdpi.com/article/10.3390/insects12070635/s1, Table S1. Statistical results of *Xenorhabdus nematophila* (Xn) assay (Figure 1). Pairwise comparisons of SWD mortality by Tukey test between different Xn concentrations at 24 and 48 h (A), and between different times within the same Xn concentration (reported as CFU/mL) (B). Significant p values (*p* < 0.05) are indicated in bold italic and highlighted in yellow. C: Control = 0 CFU/mL. Table S2. Statistical results of bioinsecticides administration assays (both single—Figure 2—and combined—Figure 3—treatments). Pairwise comparisons of SWD mortality by Tukey test between different times within the same treatment (A) and between different treatments at 16, 24, 32, and 48 h (B). Significant p values (*p* < 0.05) are indicated in bold italic and highlighted in yellow. Bt: *Bacillus thuringiensis*, Xn: *X. nematophila*, Sec: Xn secretions, C: Control, t_0_: concurrent administration, t_16_: time-shifted administration.
