# Supplementary material for: Drosophila suzukii Susceptibility to the Oral Administration of Bacillus thuringiensis, Xenorhabdus nematophila and Its Secondary Metabolites
Source: Insects. 2021 Jul 13;12(7):635. doi: 10.3390/insects12070635 (PMC8305655; doi:10.3390/insects12070635)
Supplement: Supplementary file 1 [file insects-12-00635-s001.zip › insects-1281160-supplementary.pdf]

SUPPLEMENTARY MATERIAL

Table SM1. Statistical results of Xn assay (Fig. 1). Pairwise comparisons of SWD mortality by Tukey test between different Xn concentrations at 24 and 48 h (A), and between different times within the same Xn concentration (expressed as CFU/ml) (B). Significant p values ($p<0.05$) are indicated in bold italic and highlighted in yellow colour. C: control.

**A) Comparisons between different Xn concentrations at the same time**							
**Time**	**Concentration**	**Q-value**	**p-value**	**Time**	**Concentration**	**Q-value**	**p-value**
24h	C-10^5	0.93	0.998	48h	C-10^5	0.93	0.998
	C-10^7	6.33	***0.002***		C-10^7	9.91	***0.0001***
	C-10^9	14.62	***0.0001***		C-10^9	19.17	***0.0001***
	10^5-10^7	5.40	***0.010***		10^5-10^7	8.98	***0.0001***
	10^5-10^9	13.69	***0.0001***		10^5-10^9	18.24	***0.0001***
	10^7-10^9	8.29	***0.0001***		10^7-10^9	9.26	***0.0001***
**B) Comparisons between different times within the same Xn concentration**							
**Concentration**	**Time**	**Q-value**	**p-value**	**Concentration**	**Time**	**Q-value**	**p-value**
C	24h-48h	0.93	0.998	10^7	24h-48h	4.50	0.052
10^5		0.93	0.998	10^9		5.48	***0.009***

Table SM2. Statistical results of bioinsecticides administration assays (both single - Fig. 2 - and combined - Fig. 3 - treatments). Pairwise comparisons of SWD mortality by Tukey test between different times within the same treatment (A) and between different treatments at 16, 24, 32 and 48 h (B). Significant p values ($p<0.05$) are indicated in bold italic and highlighted in yellow colour. Bt: *Bacillus thuringiensis*; Xn: *Xenorhabdus nematophila*; Sec: *X. nematophila* secretion; C: control

**A) Comparisons between different times within the same treatment**							
**Treatment**	**Time**	**Q-value**	**p-value**	**Treatment**	**Time**	**Q-value**	**p-value**
Bt	C-16h	11.30	***3.08E-05***	Bt/Xn_t_0	C-16h	21.03	***3.08E-05***
	C-24h	14.97	***3.08E-05***		C-24h	21.84	***3.08E-05***
	C-32h	16.70	***3.08E-05***		C-32h	22.04	***3.08E-05***
	C-48h	19.02	***3.08E-05***		C-48h	22.25	***3.08E-05***
	16h-24h	3.67	0.769		16h-24h	0.81	1
	16h-32h	5.41	***0.048***		16h-32h	1.01	1
	16h-48h	7.72	***5.33E-05***		16h-48h	1.22	1
	24h-32h	1.73	1		24h-32h	0.20	1
	24h-48h	4.05	0.554		24h-48h	0.41	1
	32h-48h	2.32	1		32h-48h	0.20	1
Xn	C-16h	8.10	***3.53E-05***	Bt/Sec_t_0	C-16h	17.20	***3.08E-05***
	C-24h	10.51	***3.08E-05***		C-24h	25.50	***3.08E-05***
	C-32h	13.41	***3.08E-05***		C-32h	33.32	***3.08E-05***
	C-48h	15.57	***3.08E-05***		C-48h	-	-
	16h-24h	2.41	0.999		16h-24h	8.30	***3.26E-05***
	16h-32h	5.31	0.060		16h-32h	16.13	***3.08E-05***
	16h-48h	7.47	***9.42E-05***		16h-48h	-	-
	24h-32h	2.91	0.983		24h-32h	7.83	***4.54E-05***
	24h-48h	5.06	0.107		24h-48h	-	-
	32h-48h	2.15	1		32h-48h	-	-
Sec	C-16h	3.76	0.722	Bt/Xn_t_{16}	C-16h	10.21	***3.08E-05***
	C-24h	8.13	***3.48E-05***		C-24h	12.88	***3.08E-05***
	C-32h	10.83	***3.08E-05***		C-32h	18.75	***3.08E-05***
	C-48h	11.42	***3.08E-05***		C-48h	23.11	***3.08E-05***
	16h-24h	4.36	0.374		16h-24h	2.67	0.996
	16h-32h	7.07	***0.0003***		16h-32h	8.54	***3.14E-05***
	16h-48h	7.66	***5.97E-05***		16h-48h	12.90	***3.08E-05***
	24h-32h	2.70	0.995		24h-32h	5.87	***0.014***
	24h-48h	3.30	0.917		24h-48h	10.23	***3.08E-05***
	32h-48h	0.59	1		32h-48h	4.36	0.375
				Bt/Sec_t_{16}	C-16h	9.38	***3.08E-05***
					C-24h	14.42	***3.08E-05***
					C-32h	22.72	***3.08E-05***
					C-48h	24.97	***3.08E-05***
					16h-24h	5.04	0.111
					16h-32h	13.35	***3.08E-05***
					16h-48h	15.59	***3.08E-05***
					24h-32h	8.31	***3.26E-05***
					24h-48h	10.55	***3.08E-05***
					32h-48h	2.25	1

**B) Comparisons between different treatments at the same time**							
**Time**	**Treatment**	**Q-value**	**p-value**	**Time**	**Treatment**	**Q-value**	**p-value**
	Bt-Xn	0.66	1		Bt-Xn	0.56	1
	Bt-Sec	5.82	***0.016***		Bt-Sec	4.15	0.493
	Bt-Bt/Xn_t_0	9.73	***3.08E-05***		Bt-Bt/Xn_t_0	5.34	0.056
	Bt-Bt/Xn_t_{16}	0.22	1		Bt-Bt/Xn_t_{16}	2.91	0.983
	Bt-Bt/Sec_t_0	6.76	***0.0009***		Bt-Bt/Sec_t_0	17.48	***3.08E-05***
	Bt-Bt/Sec_t_{16}	0.20	1		Bt-Bt/Sec_t_{16}	7.74	***5.20E-05***
	Xn-Sec	6.47	***0.002***		Xn-Sec	4.72	0.210
	Xn-Bt/Xn_t_0	9.08	***3.08E-05***		Xn-Bt/Xn_t_0	4.78	0.188
	Xn-Bt/Xn_t_{16}	0.88	1		Xn-Bt/Xn_t_{16}	2.34	1.000
	Xn-Bt/Sec_t_0	6.10	***0.007***		Xn-Bt/Sec_t_0	16.92	***3.08E-05***
16h	Xn-Bt/Sec_t_{16}	0.86	1	32h	Xn-Bt/Sec_t_{16}	7.17	***0.0002***
	Sec-Bt/Xn_t_0	15.55	***3.08E-05***		Sec-Bt/Xn_t_0	9.49	***3.08E-05***
	Sec-Bt/Xn_t_{16}	5.59	***0.030***		Sec-Bt/Xn_t_{16}	7.06	***0.0003***
	Sec-Bt/Sec_t_0	12.57	***3.08E-05***		Sec-Bt/Sec_t_0	21.63	***3.08E-05***
	Sec-Bt/Sec_t_{16}	5.61	***0.028***		Sec-Bt/Sec_t_{16}	11.89	***3.08E-05***
	Bt/Xn_t_0-Bt/Xn_t_{16}	9.96	***3.08E-05***		Bt/Xn_t_0-Bt/Xn_t_{16}	2.43	0.999
	Bt/Xn_t_0-Bt/Sec_t_0	2.97	0.9769		Bt/Xn_t_0-Bt/Sec_t_0	12.14	***3.08E-05***
	Bt/Xn_t_0-Bt/Sec_t_{16}	9.94	***3.08E-05***		Bt/Xn_t_0-Bt/Sec_t_{16}	2.40	0.999
	Bt/Xn_t_{16}-Bt/Sec_t_0	6.98	***0.0004***		Bt/Xn_t_{16}-Bt/Sec_t_0	14.57	***3.08E-05***
	Bt/Xn_t_{16}-Bt/Sec_t_{16}	0.02	1		Bt/Xn_t_{16}-Bt/Sec_t_{16}	4.83	0.170
	Bt/Sec_t_0-Bt/Sec_t_{16}	6.96	***0.0005***		Bt/Sec_t_0-Bt/Sec_t_{16}	9.74	***3.08E-05***
	Bt-Xn	0.61	1		Bt-Xn	0.40	1
	Bt-Sec	5.12	0.092		Bt-Sec	5.88	***0.014***
	Bt-Bt/Xn_t_0	6.87	***0.0006***		Bt-Bt/Xn_t_0	3.23	0.935
	Bt-Bt/Xn_t_{16}	1.23	1		Bt-Bt/Xn_t_{16}	4.95	0.134
	Bt-Bt/Sec_t_0	11.39	***3.08E-05***		Bt-Bt/Sec_t_0	-	-
	Bt-Bt/Sec_t_{16}	1.17	1		Bt-Bt/Sec_t_{16}	7.67	***5.92E-05***
	Xn-Sec	4.52	0.297		Xn-Sec	6.28	***0.004***
	Xn-Bt/Xn_t_0	7.48	***9.07E-05***		Xn-Bt/Xn_t_0	2.83	0.989
	Xn-Bt/Xn_t_{16}	0.62	1		Xn-Bt/Xn_t_{16}	4.55	0.279
	Xn-Bt/Sec_t_0	12.00	***3.08E-05***		Xn-Bt/Sec_t_0	-	-
24h	Xn-Bt/Sec_t_{16}	1.77	1	48h	Xn-Bt/Sec_t_{16}	7.27	***0.0002***
	Sec-Bt/Xn_t_0	11.99	***3.08E-05***		Sec-Bt/Xn_t_0	9.10	***3.08E-05***
	Sec-Bt/Xn_t_{16}	3.89	0.648		Sec-Bt/Xn_t_{16}	10.83	***3.08E-05***
	Sec-Bt/Sec_t_0	16.51	***3.08E-05***		Sec-Bt/Sec_t_0	-	-
	Sec-Bt/Sec_t_{16}	6.29	***0.004***		Sec-Bt/Sec_t_{16}	13.54	***3.08E-05***
	Bt/Xn_t_0-Bt/Xn_t_{16}	8.10	***3.53E-05***		Bt/Xn_t_0-Bt/Xn_t_{16}	1.72	1
	Bt/Xn_t_0-Bt/Sec_t_0	4.52	0.295		Bt/Xn_t_0-Bt/Sec_t_0	-	-
	Bt/Xn_t_0-Bt/Sec_t_{16}	5.71	***0.022***		Bt/Xn_t_0-Bt/Sec_t_{16}	4.44	0.334
	Bt/Xn_t_{16}-Bt/Sec_t_0	12.62	***3.08E-05***		Bt/Xn_t_{16}-Bt/Sec_t_0	-	-
	Bt/Xn_t_{16}-Bt/Sec_t_{16}	2.40	0.999		Bt/Xn_t_{16}-Bt/Sec_t_{16}	2.72	0.994
	Bt/Sec_t_0-Bt/Sec_t_{16}	10.22	***3.08E-05***		Bt/Sec_t_0-Bt/Sec_t_{16}	-	-
